# Circling the undefined—A grounded theory study of intercultural consultations in Swedish primary care

**DOI:** 10.1371/journal.pone.0203383

**Published:** 2018-08-30

**Authors:** Erica Rothlind, Uno Fors, Helena Salminen, Per Wändell, Solvig Ekblad

**Affiliations:** 1 Culture Medicine, Department of Learning, Informatics, Management and Ethics, Karolinska Institutet, Stockholm, Sweden; 2 Department of Computer and Systems Sciences, Stockholm University, Stockholm, Sweden; 3 Department of Neurobiology, Care Sciences and Society, Division of Family Medicine and Primary Care, Karolinska Institutet, Huddinge, Sweden; 4 Academic Primary Health Care Centre, Stockholm County Council, Stockholm, Sweden; Universiteit van Amsterdam, NETHERLANDS

## Abstract

Well-functioning physician-patient communication is central to primary care consultations. An increasing demand on primary care in many countries to manage a culturally diverse population has highlighted the need for improved communication skills in intercultural consultations. In previous studies, intercultural consultations in primary care have often been described as complex for various reasons, but studies exploring physician-patient interactions contributing to the understanding of why they are complex are lacking. Therefore, the aim of this study was to explore intercultural physician-patient communication in primary care consultations, generating a conceptual model of the interpersonal interactions as described by both the patients and the physicians. Using grounded theory methodology, 15 residents in family medicine and 30 foreign-born patients, the latter with Arabic and Somali as native languages, were interviewed. The analysis generated a conceptual model named circling the undefined, where a silent agreement on issues fundamental to the core of the consultation was inadequately presumed and the communicative behaviors used did not contribute to clarity. This could be a possible contributory cause of the perceived complexity of intercultural consultations. Identifying what takes place on an interpersonal level in intercultural consultations might be a first step towards building a common ground for increased mutual understanding, thereby bringing us one step closer to sharing, rather than circling the undefined.

## Introduction

Primary care is a key player in improving health equality in the population; the consultation being essential, since inadequate physician-patient communication has been linked to lower quality of care [1–4].

Demographic changes, not least as a result of recent years’ historically large migratory flows, has led to an increasing demand on health care worldwide, as well as on the individual physician, to be able to manage a culturally diverse population [1, 5, 6]. Providing culturally appropriate care is however not a recent phenomenon, the idea was introduced already in the 1950’s, and is well recognized today [7]. Likewise, the importance of good communication skills in building rapport with the patient is well established [8]. However, views on how to provide culturally appropriate care, as well as how consultations should be performed in order to foster an alliance with the patient have changed over time—there is still an ongoing discussion [9, 10]. Patient-centeredness and cultural competence are two concepts that are central in this context.

Patient-centered consultation techniques are currently widely used in Scandinavia, as well as in most English-speaking countries [11, 12]. Patient-centeredness is a concept emphasizing the importance of seeing the patient as a unique person [13]. It also stresses an increased patient engagement, shared responsibility, and the significance of understanding the patients’ view of their medical problems [10, 14]. However, this might pose an additional challenge in intercultural consultations, or physician-patient communication across cultures, where there are potential barriers to mutual understanding such as not sharing the same language or cultural backgrounds [15, 16].

The concept of culture is complex and a full account is beyond the scope of this paper, but a definition agreed useful in this context is: “Culture is a socially transmitted pattern of shared meanings by which people communicate, perpetuate and develop their knowledge and attitudes about life. An individual’s cultural identity may be based on heritage as well as individual circumstances and personal choice and is a dynamic entity” [17].

In order to attend to cultural differences in a health-care setting, the concept of cultural competence has evolved [7, 18, 19]. Cultural competence is a broad and complex concept where no universal definition exists [20, 21] but the following is used: “Cultural and linguistic competence is a set of congruent behaviors, attitudes, and policies that come together in a system, agency, or among professionals that enables effective work in cross-cultural situations” [20]. Literature on how to incorporate cultural competence in primary care practice is limited [22]. Previous studies also show that physicians in general tend to focus on generic communication skills, such as listening and conveying empathy, rather than taking intercultural perspectives into account; to a certain degree maybe failing to see to what extent cultural beliefs might influence health-related issues and communication [23, 24].

Barriers to interpersonal communication have been a recurring theme in previous qualitative as well as quantitative studies on intercultural consultations in primary care [25–28]. Intercultural consultations are often perceived by physicians as complex [25, 26, 29, 30] and stressful, partly due to lack of knowledge, confidence and skills [31–33]. Even when cultural differences have been central to the consultation, there has among physicians been a tendency to avoid addressing these issues [28]. A perceived lack of knowledge has also been linked to a limited motivation to engage in intercultural consultations [24, 31, 32].

A systematic review exploring patients’ experiences of intercultural consultations in primary care found that patients also perceived communicating with physicians challenging [34]. Contributing factors were identified and classified in the following categories: language barriers, discrimination, differences in values and beliefs, and acculturation issues [34].

The way in which so called system-related factors are perceived as barriers to care in an intercultural context has also been described; these include, for example, complicated referral systems, long waiting times and limited access to professional interpreters [30, 34, 35]. Not having sufficient access to professional interpreters might result in the reliance on informal interpreters, such as family and friends, who often assume multiple roles in the consultation such as culture-brokers, the patients advocate and care-givers [36, 37]. In addition, using informal interpreters might increase the risk of significant medical information being omitted, since it has been shown that about half of the utterances in an average consultation are not translated [37]. The quality of the consultation is likely improved with the use of professional interpreters [38, 39] but issues of confidentiality, trust, multiple roles and misinterpretations still remain [39–41].

In summary, intercultural consultations are complex as descriptive methods of analysis conclude. There is however a lack of studies aiming to generate conceptual models or theoretical frameworks contributing to the understanding of *why* this might be the case. To the best of our knowledge, studies incorporating the perspectives of patients and physicians in a combined analysis are also lacking. This demands more attention since the interaction between the two constitutes the foundation of the consultation in primary care.

Therefore, this study aims to explore intercultural physician-patient communication in primary care consultations, generating a conceptual model of the interpersonal interactions as described by both the patients and the physicians, using a grounded theory methodology taking both perspectives into account.

## Methods

The study was conducted using the qualitative method of grounded theory (GT) as outlined by Charmaz and originally developed by Glaser and Strauss [42, 43]. The grounded theory approach was employed in order to develop a conceptual model, from a bottom-up perspective. The study builds on the theoretical perspective of symbolic interactionism, with the fundamental assumption of social interaction as a base for understanding reality and the self; reality is contextual and socially constructed and human actions are thus based on constructed meanings [42]. Language and communication are essential to interaction. Cultural systems of value are dynamic concepts created in social interaction and key components of *all* consultations. However, in intercultural consultations, they might require extra consideration and sometimes provide an additional challenge.

### Setting and context

The increase in immigration to Sweden in recent years has highlighted the need for improved integration, with access to equal health care being crucial [2, 44]. The Swedish health-care system is tax funded and, in accordance with the Health and Medical Services Act, committed to providing equitable health care based on medical need; the main objective being a good health on equal terms for all individuals, regardless of social or economic status [45]. However, there is a growing concern regarding what seems to be an increasing difference in access to health care, where certain groups, such as migrants, are at risk of disadvantage [46–48]. With various primary care reforms initiated in 2007, allowing primary care centers (PCCs) to privatize, the average number of primary care visits increased. However, due to simultaneous changes in the reimbursement system it seems that more affluent groups, with a lower burden of disease, might have benefited on account of patients with more complex needs [48, 49]. In Sweden, primary care has responsibility for providing the general population with basic medical care, regardless of their socio-economic status, and it is the first point of contact for the majority of individuals seeking medical care, including migrants [47].

PCCs employ specialists (in family medicine) and residents (postgraduate physician trainees in family medicine), as well as nurses and sometimes other paramedical professionals, such as psychologists and physiotherapists [47]. A residency in family medicine in Sweden includes five years of full-time working in primary care under the supervision of a specialist. Patients are free to choose their provider of primary care, since the implementation of the so-called Primary Care Choice Reform in 2008 [50]. Each PCC is thus responsible for the patients they have listed instead of those living in the surrounding geographical area [47, 48]. The PCCs where the residents worked and the PCCs where participating patients were listed, were located in different parts of Stockholm County Council and varied in size, demographics and socio-economic characteristics, but they were all part of the same reimbursement system and had the same access to secondary or tertiary care. Due to logistic difficulties the residents did not work at the PCCs from where the patients where included.

### Recruitment and description of participants

A total of 15 residents in family medicine and 30 foreign-born patients were included. Theoretical sampling was applied when recruiting the participants. In accordance with the methodology of GT, theoretical sampling is an ongoing process in which recruitment of participants takes place continuously and in parallel with the analysis in order to achieve theoretical saturation of the emerging categories [42]. Residents were chosen as our target group, rather than specialists, since they are still in training, thus we expected them to provide us with more substantive accounts of the complexity, making areas of improvements easier to identify.

The residents were initially contacted using an e-mail list provided by the regional center for resident education in primary care. The inclusion criterion was an ongoing residency in family medicine, and there were no exclusion criteria.

The patients were initially invited to participate via written information distributed by staff working at three different PCCs in Stockholm. All patients included were part of the Swedish migrant population and had obtained Swedish residence permits. Exclusion criteria were being under the age of 18 or over 65. Due to their strained circumstances asylum-seekers and undocumented migrants were also excluded.

The characteristics of the participants are outlined in Tables 1 and 2.

**Table 1:** Characteristics of the residents who participated in the interviews concerning intercultural consultations in primary care (*n* = 15).

Characteristic	Number (%)	Mean (SD)
Gender:		
• Men	10 (67)	
• Women	5 (33)	
Medical degree from:		
• Sweden	8 (53)	
• Other country	7 (47)	
Years in profession		4.9 (1.6)

**Table 2:** Characteristics of the patients who participated in the interviews concerning intercultural consultations in primary care (*n* = 30).

Characteristic	Number (%)	Mean (SD)
Gender:		
• Men	15 (50)	
• Women	15 (50)	
Level of education:		
• Primary	12 (40)	
• Secondary	9 (30)	
• Higher	9 (30)	
Country of origin:		
• Afghanistan	4 (13)	
• Iraq	2 (7)	
• Somalia	18 (60)	
• Syria	6 (20)	
Years living in Sweden		9.6 (6.9)
Age		41.6 (9.1)

Including foreign-born residents having received their medical education abroad was deemed important since they constitute a great part of the body of physicians in Sweden today [51].

### Ethics

All informants were given verbal and written information on the purpose of the study. The verbal information was given in Swedish or, when needed, given in the native language by staff at the recruiting PCCs, and also a second time at the interview (through the use of a professional interpreter if needed). The written information for the patients was translated by professional translators into their respective native languages. Even though the majority of the patients declined the offer of an interpreter during the interviews, we found it important to ensure they were all given written information in their native language on confidentiality and the voluntary nature of participation, with the right to decline or withdraw from the study at any given moment without the need to explain why. Informed consent was given in writing by each participant, when needed in their native language. The transcripts were anonymized.

The study was approved by the Regional Ethical Review Board in Stockholm (2015/1228-31/5 and 2016-2308-32).

### Data collection and analysis

Data was collected by means of individual and focus group interviews. All interviews took place in Stockholm between September 2015 and September 2017, in locations chosen by the participants; these included meeting facilities provided by local libraries, Karolinska Institutet or in some instances the PCCs.

In total, three focus groups consisting of four to seven participants, and 31 individual interviews were carried out. The residents were interviewed individually and the patients were interviewed both individually and in focus groups. Focus groups with patients were used in addition to individual interviews to explore if new information would emerge through the interaction of the informants [52], which was not the case although a richer account was given in general.

All focus groups were led by the first author who acted as moderator, while the last author acted as observer. Master-level medical students conducted the individual interviews, with both residents and patients, under close supervision by the first and last authors. In qualitative methods, the researcher is a part of the research process bringing prior experiences and knowledge “to the table”. The first author, being a specialist in family medicine, and the last author a licensed psychologist and researcher with focus on intercultural health care, enable theoretical sensitivity, but also represent a risk of bias, and we hypothesized that the authors’ positions might also be a hindering factor preventing the informants speaking freely and bringing forth possible complaints or insecurities. This was the main rationale for medical students being involved in the interviews.

Initial interview guides with open-ended questions were developed, one for the residents and one for the patients. Questions were formulated with the intention of illustrating the patients′ and the residents′ respective experiences of intercultural consultations in primary care, with the main focus for all interviews and focus groups being physician-patient communication. The questions were formulated with slight variations for the two groups but the same aspects were covered; these included experiences of more and less satisfactory consultations, trust-building and how to reach mutual understanding. Throughout we continuously asked for differences and similarities between inter- and intracultural consultations. Follow-up questions focusing on behaviors were frequently used. Interviews were conducted using a semi-structured interview technique, which enables the interviewer to explore new areas of interest emerging during the interview. The data collection and analysis were carried out in parallel, and in accordance with GT as preliminary concepts and categories emerged the interview guides were revised by the first and last author. Preliminary categories emerging early on were for example ambiguities regarding roles and responsibilities, this was then penetrated further.

Each interview was audiotaped, transcribed verbatim, and analyzed prior to the next. The interviews were transcribed by the respective interviewer. In the cases where the first author did not interview and thus did not transcribe, the audiotape was listened to by the first author. Interviews were carried out until theoretical saturation was achieved, that is when new information no longer adds new theoretical insights [42].

Each patient was given the opportunity to use an interpreter. However, the majority declined and in the end only 11 of the 30 patients included were in need of an interpreter. Parts of the interviews where interpreters were used were reviewed by independent professional translators in order to verify the accuracy of the interpretations. In general, the interpretations were deemed accurate with the exception of the interpreters sometimes shortening the informants’ answers, although according to the translators this did not change the content.

In accordance with GT as outlined by Charmaz [42] the data analysis used initial, focused coding and categorizing. Initial coding was carried out by the first and last author generating rudimentary codes. During focused coding the initial codes were tested against a gradually increasing amount of data generating substantive preliminary categories. Through categorizing the analysis was raised from a descriptive to a more theoretical level and a concept was created and defined. One example being the quote: *“The physician should never ask questions that aren’t to do with the patient’s problems”* which generated the following coding steps: not accepting questions “out-of-context”–selective story-sharing—fragmentizing the story. By constantly returning to the original coding and data throughout the analysis, the final concept was grounded in the data. Memos were written continuously throughout the research process in accordance with the method. The analysis was conducted in Swedish, this being the native-language of the research team, and was continuously discussed in seminars with the co-authors.

The relevance of the results was checked by the use of key informants and additional focus groups. The key informants were two senior physicians with a deeper knowledge of the area through a vast experience in working with migrant patients. One focus group consisted of five patients with migratory backgrounds similar to the informants and one focus group consisted of eight residents in family medicine working at various PCCs in Stockholm. In summary, they considered the results relevant and the residents found the conceptual model applicable to their work. No concepts were changed. The informants were also invited to comment on the results.

## Results

### Circling the undefined—A conceptual model

Difficulty in dealing with the undefined emerged as a main concern in the analysis. Both residents and patients seemed to experience this. The undefined affecting the interpersonal communication in intercultural consultations incorporates an inadequately presumed silent agreement on the format and/or content of the consultation, and on the fundamental views on what constitute health and illness, all contributing to a predominance of symptoms and concerns left without a “sufficient” or shared explanation. *“It’s a huge source of frustration when you*, *because it can sort of happen*, *that in a consultation you notice that it’s going to happen here*, *that she [the patient] will mention loads of symptoms[…]it’s like a cloud of symptoms being hurled at you…”(res1)*.

Dealing with the undefined was recognized by the residents as part of their work in family medicine, nonetheless being perceived as challenging and time-consuming. *“They [foreign-born patients] do not really know how health care works and what you do in*, *for example a consultation*. *So that is also a challenge*.*” (res7) “You have a ten minute appointment for a child with tonsillitis*, *and then the whole family shows up*, *and they all have different symptoms [they want to discuss]*. *That happens a lot*.*” (res5) “It is different from Swedish [patients]*, *like the whole concept of illness and treatment*. *“(res12)*.

Within the same culture there is often a sufficient level of agreement on the aspects incorporated in the undefined, thus there is no need for clarification in the individual consultation. However, when basic views on these areas differ substantially and this is not recognized, it is likely to create misunderstandings adding to the complexity of the intercultural consultation. *“So often this thing about what is a disease and what is not a disease that is something causing misunderstandings between the physician and the patient*.*” (FGI1pat3)* The categories described in our model are behaviors applied by residents and mirrored by patients, that might serve a function in some consultations, for example, offer a simplified explanation or save time. However, in intercultural consultations they will not bring about a shared understanding, but rather lead to a circling of the undefined, resulting in the core of the problem not being sufficiently addressed.

**Fig 1:**
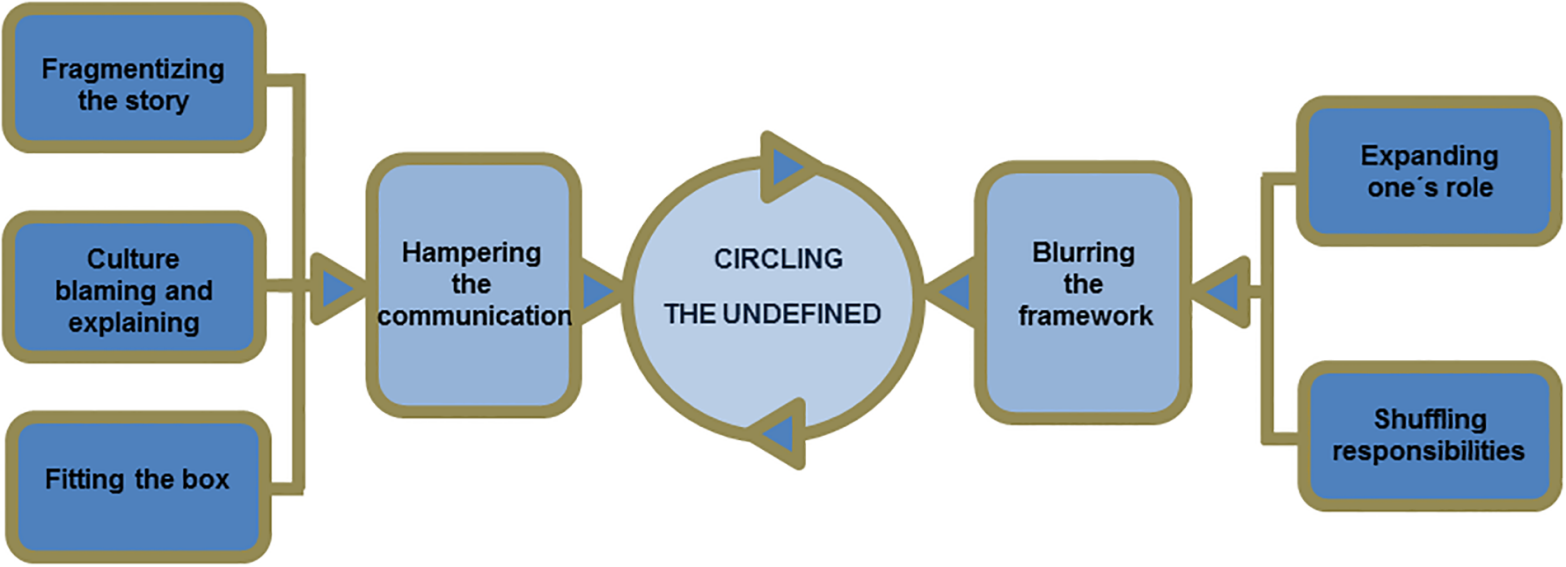
A conceptual model describing how residents and patients through their behaviors keep circling the undefined in intercultural consultations.

Two main categories and five sub-categories emerged in the analysis of the resident- and the patient-interviews. In different ways, although not explicitly intended, they seemed to lead to a circling of the undefined without getting closer to the core of the problem. The categories are described separately below and they form the basis for our conceptual model: circling the undefined, as illustrated in Fig 1.

### 1. Hampering the communication

If significant information is not shared the undefined will not be clarified. Communication is found to be hampered in different ways as outlined below in three sub-categories. Fragmentizing the story leads to a shattered holistic view. Preconceptions are applied as part of culture blaming and explaining. Fitting the box involves assuming adequate knowledge.

#### 1.1 Fragmentizing the story

Fragmentizing the story means restricting history taking; in worst case it resulted in a shattered holistic view where only fragments of the patient’s history were made available to the resident. This behavior was described as a technique of dealing with what the residents perceived as being too limited a time available for the consultation.

In intercultural consultations time limitation was perceived as particularly problematic since, in addition to language difficulties there could be a different communication style, described by the residents as often elusive and unstructured. This sometimes resulted in difficulties assessing the severity of described symptoms. *“When you express yourself in a different way it is more difficult to assess if it is anything serious or not*, *and then when there are a lot of symptoms you tend to think ‘no*, *this is probably not that serious*, *it is like too much*, *and if all of that would be signs of somatic disease you wouldn’t even be able to walk in here*.*” (res1)*.

Fragmentation became a way in which to try to create structure and sometimes also, for better or for worse, to simplify a complex account. There was however ambivalence among the residents regarding this strategy; it might facilitate a more manageable work situation but at the cost of losing the overall perspective. *“But you could also assess the stomach pain and the headache separately and say*: *‘There is nothing wrong with your stomach and there is nothing wrong with your head*, *end of story’ and be satisfied*. *We are all different*, *and who knows maybe that is sometimes not such a bad way of doing things*.*”(res2)*.

The patients also described how they often chose to be quite selective in sharing their story. Reasons could be, for example, fear of a lack of confidentiality, experiencing encroachment into their private life when asked questions about for example family, and feeling that questions were asked out of context. *“The physician should never ask questions that aren’t to do with the patient’s problems*. *They should keep to the problem you have*.*”(pat4)* However, patients having lived for a longer period of time in Sweden described how they had become accustomed to questions from physicians which they initially felt were intrusive and compromising their integrity. *“From the beginning I felt he [the physician] was stepping into my private life*, *but now I’ve gotten used to it and I don’t think it’s strange anymore*.*” (pat6)*.

#### 1.2 Culture blaming and explaining

Culture blaming and explaining means ascribing various traits or behaviors of the patient, or the physician, based on their belonging to a certain culture differing from one’s own, implying that all members of that group share the same traits. Behaviors or symptom-manifestations can then be explained by, or blamed on culture, minimizing the need for a more profound assessment. This was a strategy that both patients and residents applied with what seemed to be varying levels of awareness.

*“It’s a cultural thing”* was a recurring phrase used by both patients and residents when referring to behavioral aspects of the consultation which they did not understand. This included for example the ability (or disability) to express empathy, the tone and volume of voice, whether or not to trust a physician asking colleagues for advice and how much value to place on a physical examination.

One consequence of culture blaming and explaining was described by the patients as a perceived sense of discrimination through being assessed as part of a group instead of individuals. One patient gave the following example: *“‘Why do you Somalis have pain in either your left or right arm*? *What are you DOING with your arms*?*’ I said*: *‘are you asking me or all Somalis*? *I’m the only one here now*. *I don’t know if it’s the left or right arm with the rest of them*.*’ He [the physician] said*: *‘we had a meeting at the hospital*, *and we discussed the fact that ALL Somali women had pain in either their left or right arms*.*’” (FGI2pat4)*.

While residents in the interviews expressed an explicit reluctance to generalize about foreign-born patients, the existence of prejudices was admitted and the difficulty to find time to reflect on this in day-to-day work was a concern. *“I think you just work*, *without thinking too much… of course you have your prejudices lurking below the surface*, *and it is important to talk about them*.*” (res1)*.

#### 1.3 Fitting the box

Fitting the box refers to how the residents seemed to work with a mind-set of well-defined “boxes” in terms of guidelines, care programs and diagnostic manuals for what is common and expected to present at an average PCC. In one of these boxes each patient is sooner or later “being made” to fit. In intercultural consultations the risk for “a bad fit” is more evident, due to, for example, different interpretations or understandings of various concepts and the prevalence of different diseases varying across populations. Fitting the box could also become an indirect way of showing the patient what is accepted in a specific cultural context. It was, with self-distance referred to as a process of integration by one of the residents: *“It is a process of integration where the patients are forced to adapt to fit into our diagnostic manuals*.*” (res8)*.

Fitting the patient into existing diagnostic manuals was something that some residents expressed concern regarding. Could it be considered good practice to place the patient in a diagnostic category when the concept might have a different meaning to them? One example discussed was the non-universal diagnosis “maladaptive stress reaction”. The patients expressed how they felt this diagnosis was a way to dismiss them: *You have an appointment with a doctor*, *maybe only 5–10 minutes*, *and he says*: *‘how are you*?*’ and then he turns around looking at his computer*. *You keep talking and then [he] says*: *‘ok*, *so I think you have a stress-reaction’*. *Oh please*!*” (FGI2pat6)*.

In addition to indirectly imposing more or less relevant or understandable concepts on the patient, fitting the box could also result in an increased risk of physician’s delay. Both patients and residents gave examples of this in the interviews. With infections, for instance, it might involve failing to take into account a patient’s stay in their home country, thus missing to test for pathogens not included in the standardized care programs. Another example discussed was female genital mutilation. *“I have had female patients who had undergone circumcision… who said nothing about it themselves*, *but who came in for other problems that are related to it… and I had not considered that it could have been the case*.*”(res6)* For a lot of residents, even though they might possess theoretical knowledge of the phenomenon, the idea of female genital mutilation is still outside their well-defined boxes, and therefore easily overlooked.

One patient summarized it with the following quote: *“Dare to think ‘outside the box’*, *sometimes*, *not every day*, *I know that you have something to follow*, *some guidelines… But sometimes*…*” (FGI1pat2)*.

### 2. Blurring the framework

Through expanding one′s role and shuffling responsibilities the framework for the consultation becomes unclear. Thus the conditions are favorable for the undefined to remain so, rather than help clarifying it. The two sub-categories are presented below.

#### 2.1 Expanding one’s role

The residents reported that in intercultural consultations they often became something more than just a physician. Expanding one’s role involves the resident trying to take on functions of other professions (or bodies), or being assumed to possess these functions by the patients. This seemed to occur more or less subconsciously and involuntarily. Examples given by the residents of what the physician’s role could be expanded to include were: social representative, social welfare officer, psychologist, Swedish teacher, “a walking encyclopedia”, coach and advocate in dealings with the authorities.

This could affect the consultation in different ways; one being spending a lot of time on non-medical issues. This could include everything from drawing maps to the nearest pharmacy, to more complicated and emotionally demanding tasks such as having a supportive role in the patient’s life.”*In some cases my experience is that I’ve become a pretty large part of their new lives here*, *that maybe I’m the only one they are in touch with besides someone from the Swedish Migration Agency*. *It seems to fill some other need than a medical need that they want to visit quite often in the beginning; it’s like some sort of safety*.*” (res6)*.

Role expansion also affected the consultation in that it could be a potential source of confusion and conflict, since there seemed to be a recurring mismatch between what the residents recognized their role to be, and the demands they experienced coming from the patients, or via the patients from bodies of authority. *“This patient has come to see me six or seven times because he had to move from one apartment to the other and has no permanent living quarters*. *What am I supposed to do with this patient*? *Write a certificate stating ‘the patient is ill and needs an apartment’–will that make the municipal officer solve the problem*? *No*.*” (res5)* In one of the focus groups there was a discussion among the patients on how physicians declining to issue certificates (similar to the one mentioned in the quote above), could be perceived as xenophobic. *“So he [the patient] thinks the physician doesn’t want to write it… this certificate the patient needs […] and that creates a lot of… I’ve honestly talked to people who think the doctor is racist*, *that’s what I’ve seen*, *even among my friends*.*” (FGI1pat3)* From a patient perspective, a potential consequence could be a decreased level of trust, not only in the physician, but in the health care system as a whole.

Possible consequences for the individual resident could also be identified. Some of the residents expressed that the demands they felt they were under to expand their role entailed a feeling of inadequacy and powerlessness, which in the long run might result in less job-satisfaction and increased levels of work-related stress. *“It becomes like a part of your everyday work*, *recurring problems*, *which as a fellow human you naturally think of as terrible and horrible*. *But in your professional role you can’t do anything about it… that constant feeling of not doing anything good or helping in anyway*.*" (res5)*.

#### 2.2 Shuffling responsibilities

Shuffling responsibilities is a behavior which is result of a discrepancy between the patients’ and the residents’ views on where the line is drawn between their respective responsibilities. There seemed to be an expectation that this demarcation should be self-evident, requiring no explicit clarification, but this is far from always being the case; it varies over time and by societal form. Instead of establishing who takes responsibility for what, there seemed to be a tendency to hold the other party accountable—hence the concept shuffling responsibilities.

Who had the main responsibility for the patient’s health was one example where the residents’ and the patients’ views could differ: *“Physicians have a role*, *I came to you as a patient […]*, *I came because you have a lot of knowledge*, *I need help*, *need to get rid of this problem*. *You have to take over the medical side of things*, *all this medical stuff*.*”(FGI1pat1)* Coming from a culture where patient engagement is not an established concept, and where the style of communication might be more paternalistic, expecting the physician to “take over” might be adequate behavior. If the resident on the other hand is trained in an environment where a high degree of patient engagement is expected, this behavior might cause frustration; involving the patient in the treatment was for example found to be more of a challenge. *“You might think that the physician can fix a whole lot*, *perhaps without having to do anything yourself*, *you can just get some pills that can fix most things*. *I have seen a lot of that sort of thing and have to work hard to explain*, *or to get the patient to realize that they can be involved in the treatment*.*”(res6)* The consequence could be mutual dissatisfaction with the consultation and consequently maybe an inferior medical treatment, which might have been possible to avoid if a clear agreement on the concept of shared responsibilities, had been established from the beginning.

## Discussion

### Statement of principal findings

Dealing with the undefined seemed central to a successful physician-patient interaction, since in intercultural, as opposed to most intracultural consultations a sufficient consensus on the aspects included in this concept could not be assumed. In summary, an inadequately presumed silent agreement on the format and/or content of the consultation and on basic views on the concepts of health and illness contributed to a predominance of symptoms and concerns left without an explanation. Addressing the undefined, as part of the intercultural consultation, could potentially result in less misunderstandings, frustration and insecurities. However, we identified several communicative behaviors among the residents, mirrored by the patients, which (although probably unintentionally) seemed to maintain rather than resolve the undefined. The behaviors described in our first main-category, hampering communication, might inadvertently signal a wish for distance and reluctance to interact on a more profound level. Whereas potentially colliding views on roles and responsibilities described in our second main-category, blurring the framework, seemed to result in an insecure interaction. In different ways this might contribute to a negative communication climate where voicing questions and concerns (although possibly shared) are not facilitated and where differing views or ideas are not likely to emerge but rather be left unidentified. Hence, the conceptual model of circling the undefined.

### Findings in relation to previously published work

Communication is inherently cultural since our cultural belonging shape the way we interpret what is being said, how it is being said and our understanding of implicit meanings [34]. This is fundamental to consider when reflecting on interpersonal interaction in intercultural consultations; in other words *both* patients and physicians bring their respective cultural background to the consultation, thus potential difficulties arising are consequences of the interaction between the two, rather than due to the patient “having a different culture” [17]. Upholding a dual perspective might be facilitated by global mobility leading to an increasing number of physicians, in Sweden as well as internationally, coming from or being educated abroad [21, 24, 51].

It is also central not to underestimate similarities between cultures, which can be illustrated by several generic communication skills being equally valued by patients, regardless of cultural belonging; examples are attentive listening, being treated as an individual and with respect, and being granted a sufficient amount of time [53–55]. Thus, our conceptual model will be discussed both in relation to concepts pertaining to intercultural communication such cultural competence and humility, as well as in relation to patient-centering.

First however the concept of uncertainty will be mentioned in relation to our conceptual model in order to briefly outline how we distinguish it from the undefined. Uncertainty has many different constructs and a comprehensive discussion is beyond the scope of this paper. It is however often mentioned in the literature as pertaining to the physician’s diagnostic uncertainty [56–58] and as being a function of the physician’s biomedical knowledge, clinical judgement and information processing skills [59]. Emphasized is that an element of uncertainty is unavoidable in *all* consultations (irrespective of them being inter- or intracultural), this being due to the uncertainty inherent in the field of medicine itself [59]. The limited capacity of evidence based medicine to predict outcomes for the individual patient being one example [59]. Circling the undefined is a result of the physician-patient interaction in intercultural consultations and unlike uncertainty the concept is not inescapable. However, it does require clarification through communicative behaviors, inevitably taking culture into serious consideration.

Cultural competence is a concept with great impact albeit not having a universal definition, as mentioned in the introduction [20, 22, 60]. Two systematic reviews looking at the concept from different angles were published in 2016, with an agreement in the majority of the articles that cultural competence entails three major components: cultural knowledge, awareness and skills [20, 22]. One example from our results demonstrate how a seemingly basic question such as asking about family, might actually in an intercultural consultation cause perturbation if these components are lacking. In our sub-category of fragmentizing the story, one patient describes over time having learned to accept questions about family, initially considered very private. Having a basic knowledge of different worldviews might in this instance be helpful. In countries where a collectivistic worldview is predominant, the group is highly valued and protected [61]. This might, from a Swedish perspective, being a highly individualistic culture [61], seem foreign. If the physician is not aware of the implications of these different so called cultural values [62], patients fragmentizing their story when being asked questions about family might be misunderstood or mistaken for having a hidden agenda if reluctant or neglecting to answer. However, the risk of over-generalizing is important to consider when discussing the concept of cultural competence. The concept of cultural competence has been criticized for increasing the risk of the individual being overlooked, in favor of simplified stereotypical clarifications based on culture being the same as ethnicity or nationality [60]. This risk is more inherent in the so-called cultural expertise approach of cultural competence which is fact-driven and reflects biomedical teaching in that absolute truths can be discovered and learned [63]. The dominance of biomedical teaching in medical training can be said to foster medical students into a biomedical micro-culture [34].

The concept of micro-culture refers to groups within the general cultural context sharing different traditions and/or behaviors based on for example gender or profession [64]. Physicians are for example, as mentioned, trained within a dominant micro-culture of biomedicine; which has been suggested to enhance “an asymmetric physician-patient relationship” contributing to patients experiencing a sense of vulnerability and helplessness in intercultural consultations [34]. In addition, if the undefined is circled, as suggested by our conceptual model, it has the potential of further reinforcing this asymmetry in intercultural consultations, since a presumed silent agreement might result in lack of information relevant in empowering the patient. Through our conceptual model it might be possible for the individual physician to identify communicative behaviors contributing to this asymmetry and consequently through awareness and change facilitate an interaction on more equal terms. However, an asymmetric patient-physician relationship is not only maintained by the physician, it can also be sustained or enlarged by the patient as seen in our category of shuffling responsibilities where the patient expressed a wish for the physician to “take over all this medical stuff”.

Shuffling responsibilities is a communicative behavior which suggests that the aspects of patient-centering concerning patient engagement and shared decision making, mentioned in the introduction, might need to be adapted in some intercultural consultations in order to avoid mutual dissatisfaction. Adapting the style of communication to the individual patient in accordance with patient-centering, might thus in the short run involve maintaining a more asymmetric patient-physician relationship. We are thereby not suggesting that patient-centered techniques are not applicable in intercultural consultations. On the contrary, our sub-categories of culture blaming and explaining and fitting the box demonstrate what happens when patient-centering is neglected: the individual patient is hidden either in the collective or behind formal care programs.

Another concept also stressing the inclusion of the individual patient and addressing power imbalance is cultural humility [65, 66]. The physician committing to self-reflection is also a main component of the concept [65, 66]. Our sub-category of culture blaming and explaining emphasizes this need, as the risk for an ethnocentric approach is inherent in this communicative behavior. Previous studies also confirm that physicians do not usually reflect on their own cultural background and its possible influence on the consultation [23, 28].

With the interrelated concepts of cultural competence, patient-centering and cultural humility evolving alongside there has also been a call for the application of an intersectional framework in intercultural medical curricula, based on the premises that multiple social and cultural statuses such as gender, ethnicity, sexuality, level of education and social class co-exist and interact to shape the needs and experience of each individual [10, 67, 68]. By applying an intersectional approach it might also be easier to envision to what extent the undefined, as outlined in our model, might be present and in need of being addressed in the individual intercultural consultation, rather than using a “one-size-fits-all approach” with a dominant focus on culture in its more narrow sense. Through our model an intersectional approach might be facilitated by a shift in focus from identifying and understanding specific”cultural phenomena”, to behaviors applicable in a more wider sense in intercultural consultations. The conceptual model can be visualized as a malleable framework of interrelated concepts; they are not possible to rank in significance in a generalized way; for one consultation a specific behavior might be the principal cause of the undefined not being disclosed, while in another a combination of several behaviors might be the reason. However, the concepts in the framework are interrelated in that they all contribute to a less than optimal interaction, unintentionally enhancing an asymmetric patient-physician relationship instead of promoting a disclosure of the undefined. With multiple and complex intersectionalities of culture and health being present in intercultural consultations in primary care, where a holistic approach is considered a core value, some has even suggested that residents as part of their training to become specialists in family medicine need to undergo “a cultural shift”; to reorient their biomedical thinking in order to adopt to “a new cultural role as primary health care providers” [69]. In intercultural consultations our conceptual model might be a helpful tool in doing so.

### Strengths and limitations

A GT study can be evaluated using various criteria, however, the criteria of relevance, fit, workability and modifiability as outlined by Glaser are described by Charmaz as “particularly useful” [42, 70].

This study included a diverse group of informants and employed the use of both individual and focus groups interviews, resulting in rich and saturated data. The ability to identify a main concern in such a heterogeneous group emphasizes the relevance of the results. Reflecting the body of physicians working in primary care in Sweden today we found it important to also include residents having received their medical training abroad, thus adding to the heterogeneity and incorporating a wider perspective of intercultural consultations. Since the setting of the study was a multicultural environment, regardless of the residents’ origins they frequently met patients from other cultural backgrounds. Relevance was also confirmed by so-called member-checking.

Fit means the model is directly related to empirical data. Since data collection and analysis were performed in parallel, in accordance with GT methodology, the results can be considered well-grounded in the data. Thus, they reflect the perceptions of the residents and the patients.

The use of different interviewers can be regarded as a limitation. This might be outweighed by the risk of the informants being less inclined to share any negative views, had the first author, who is also a specialist in family medicine, been the only interviewer. Close collaboration and regular supervision also balanced the possible negative aspects.

The main limitation of this study was language barriers, with the consequent need for interpreters in some cases, even though the majority of the informants were proficient in Swedish. Using interpreters changes the conditions for the research interview, with the researcher having to rely on the interpreter’s skills and qualifications and data being co-constructed by three parties [71]. Just as in medical consultations the presence of an interpreter in research interviews may have an impact on issues of trust and confidentiality and consequently on what information the informant chooses to disclose [39–41, 71, 72]. The risk for misconstructions was compensated for by using only professional interpreters and applying translation-checking, previously described in the methods section. When analyzing the interviews there were not any systematic differences between the group using interpreters and the one proficient in Swedish, both groups also expressed negative and positive statements.

Other aspects pertaining to data collection that might influence the results are the location of the interview and the recruitment procedure. The participants were always given the option to choose location and to the extent possible “neutral” places were suggested. The recruitment procedure was undertaken with emphasis on confidentiality and the voluntary nature of participation in accordance with the ethical permit. When recruiting patients, the procedure also included clear information that their care would in no way be affected by their decision to participate. Choosing to recruit residents, as opposed to specialists, was an active choice as described in the methods section, this might however also have had a bearing on the result in that the complexity encountered might be more prominent. For example, a lack of continuity with the patient over a longer period of time, which residents being in the beginning of their career often have not had the opportunity to establish, is likely to contribute to a sense of complexity.

The result of a GT study is always a product of the interaction between the researchers, the informants and the data. Thus, researchers need to reflect on how their own cultural beliefs influence the data. This was continuously discussed in group sessions and through the use of memos.

Interviewing specialists and performing observations in a clinical setting would be valuable in order to gain further understanding of a complex area and to check the workability and modifiability of the constructed model. This is planned for in future studies.

### Implications

Short term: The study emphasizes the importance of focusing on interpersonal interactions in intercultural consultations and training, as well as highlights the need for interventions aimed at both residents and patients to facilitate communication. For residents, as a first step, we wish to propose an expanded capacity for self-reflection under supervision, based on an experiential learning perspective.

Long term: Through improved consultation skills, optimize the care of foreign-born patients in primary care, thus reducing the risk of inequality in health care.

## Conclusion

Intercultural consultations are complex, this study is an attempt to construct a conceptual model of interpersonal interactions contributing to the understanding of why. Understanding how communicative behaviors used might fail to address key elements of the consultations incorporated in the undefined might be a first step towards building a common ground for increased mutual understanding. Consequently, we might also be one step closer to sharing, rather than circling the undefined.

## References

[1] Next step towards more equity in health in Sweden: SOU2017:47 The Swedish Commission for Equity in Health [Internet]. [cited: 2017 Dec 12]. http://www.regeringen.se/rattsdokument/statens-offentliga-utredningar/2017/06/sou-201747.

[2] Official Report of the Swedish Government: On Equal Health: SOU2016:55 [In Swedish]. Stockholm: The Swedish Commission for Equity in Health, 2016 978-91-38-24484-5.

[3] Beck RS, Daughtridge R, Sloane PD. Physician-patient communication in the primary care office: a systematic review. The Journal of the American Board of Family Practice. 2002;15(1):25–38. Epub 2002/02/14. . 11841136

[4] van Wieringen JC, Harmsen JA, Bruijnzeels MA. Intercultural communication in general practice. Eur J Public Health. 2002;12(1):63–8. Epub 2002/04/24. . 11968523 10.1093/eurpub/12.1.63

[5] The National Board of Health and Welfare. Healthcare and dentalcare for asylumseekers and newly arrived: Final Report, October 2016 [In Swedish]. Stockholm: The National Board of Health and Welfare, 2016.

[6] Diaz E, Kumar BN. Health care curricula in multicultural societies. Int J Med Educ. 2018;9:42–4. Epub 2018/02/23. 10.5116/ijme.5a7e.bd17 . 29470179 PMC5834828

[7] Leininger MM. Transcultural nursing: concepts, theories, and practices New York: Wiley Medical; 1978.

[8] Hampton JR, Harrison MJ, Mitchell JR, Prichard JS, Seymour C. Relative contributions of history-taking, physical examination, and laboratory investigation to diagnosis and management of medical outpatients. British medical journal. 1975;2(5969):486–9. Epub 1975/05/31. . 1148666 10.1136/bmj.2.5969.486 PMC1673456

[9] Beach MC, Price EG, Gary TL, Robinson KA, Gozu A, Palacio A, et al Cultural competence: a systematic review of health care provider educational interventions. Medical care. 2005;43(4):356–73. Epub 2005/03/22. . 15778639 10.1097/01.mlr.0000156861.58905.96 PMC3137284

[10] Saha S, Beach MC, Cooper LA. Patient centeredness, cultural competence and healthcare quality. Journal of the National Medical Association. 2008;100(11):1275–85. Epub 2008/11/26. . 19024223 10.1016/s0027-9684(15)31505-4 PMC2824588

[11] Brouwers M, Rasenberg E, van Weel C, Laan R, van Weel-Baumgarten E. Assessing patient-centred communication in teaching: a systematic review of instruments. Medical education. 2017 Epub 2017/08/02. 10.1111/medu.13375 . 28762538 PMC5655924

[12] Mead N, Bower P. Patient-centred consultations and outcomes in primary care: a review of the literature. Patient education and counseling. 2002;48(1):51–61. Epub 2002/09/11. . 12220750 10.1016/s0738-3991(02)00099-x

[13] Balint E. The possibilities of patient-centered medicine. The Journal of the Royal College of General Practitioners. 1969;17(82):269–76. Epub 1969/05/01. . 5770926 PMC2236836

[14] Lewin SA, Skea ZC, Entwistle V, Zwarenstein M, Dick J. Interventions for providers to promote a patient-centred approach in clinical consultations. The Cochrane database of systematic reviews. 2001;(4):Cd003267. Epub 2001/11/01. 10.1002/14651858.cd003267 . 11687181

[15] Paternotte E, Scheele F, Seeleman CM, Bank L, Scherpbier AJ, van Dulmen S. Intercultural doctor-patient communication in daily outpatient care: relevant communication skills. Perspect Med Educ. 2016;5(5):268–75. Epub 2016/09/18. 10.1007/s40037-016-0288-y . 27638395 PMC5035277

[16] Paternotte E, van Dulmen S, van der Lee N, Scherpbier AJ, Scheele F. Factors influencing intercultural doctor-patient communication: A realist review. Patient education and counseling. 2015;98(4):420–45. Epub 2014/12/24. 10.1016/j.pec.2014.11.018 . 25535014

[17] Dogra N, Bhatti F, Ertubey C, Kelly M, Rowlands A, Singh D, et al Teaching diversity to medical undergraduates: Curriculum development, delivery and assessment. AMEE GUIDE No. 103. Medical teacher. 2016;38(4):323–37. Epub 2015/12/09. 10.3109/0142159X.2015.1105944 . 26642916

[18] Carrillo JE, Green AR, Betancourt JR. Cross-cultural primary care: a patient-based approach. Annals of internal medicine. 1999;130(10):829–34. Epub 1999/06/12. . 10366373 10.7326/0003-4819-130-10-199905180-00017

[19] Dunn AM. Culture competence and the primary care provider. Journal of pediatric health care: official publication of National Association of Pediatric Nurse Associates & Practitioners. 2002;16(3):105–11. Epub 2002/05/17. . 12015668

[20] Alizadeh S, Chavan M. Cultural competence dimensions and outcomes: a systematic review of the literature. Health & social care in the community. 2016;24(6):e117–e30. Epub 2015/10/27. 10.1111/hsc.12293 . 26499469

[21] Horvat L, Horey D, Romios P, Kis-Rigo J. Cultural competence education for health professionals. The Cochrane database of systematic reviews. 2014;(5):Cd009405. Epub 2014/05/06. 10.1002/14651858.CD009405.pub2 . 24793445 PMC10680054

[22] Watt K, Abbott P, Reath J. Developing cultural competence in general practitioners: an integrative review of the literature. BMC Fam Pract. 2016;17(1):158 Epub 2016/11/17. 10.1186/s12875-016-0560-6 . 27846805 PMC5111200

[23] Paternotte E, Scheele F, van Rossum TR, Seeleman MC, Scherpbier AJ, van Dulmen AM. How do medical specialists value their own intercultural communication behaviour? A reflective practice study. BMC medical education. 2016;16(1):222 Epub 2016/08/26. 10.1186/s12909-016-0727-9 . 27558271 PMC4997670

[24] Rosenberg E, Richard C, Lussier MT, Abdool SN. Intercultural communication competence in family medicine: lessons from the field. Patient education and counseling. 2006;61(2):236–45. Epub 2005/06/22. 10.1016/j.pec.2005.04.002 . 15967625

[25] Jensen NK, Norredam M, Priebe S, Krasnik A. How do general practitioners experience providing care to refugees with mental health problems? A qualitative study from Denmark. BMC Fam Pract. 2013;14:17 Epub 2013/01/30. 10.1186/1471-2296-14-17 . 23356401 PMC3568406

[26] Papic O, Malak Z, Rosenberg E. Survey of family physicians’ perspectives on management of immigrant patients: attitudes, barriers, strategies, and training needs. Patient education and counseling. 2012;86(2):205–9. Epub 2011/06/04. 10.1016/j.pec.2011.05.015 . 21636237

[27] Schouten BC, Meeuwesen L. Cultural differences in medical communication: a review of the literature. Patient education and counseling. 2006;64(1–3):21–34. Epub 2006/01/24. 10.1016/j.pec.2005.11.014 . 16427760

[28] Wachtler C, Brorsson A, Troein M. Meeting and treating cultural difference in primary care: a qualitative interview study. Fam Pract. 2006;23(1):111–5. Epub 2005/10/26. 10.1093/fampra/cmi086 . 16246851

[29] Kai J, Beavan J, Faull C, Dodson L, Gill P, Beighton A. Professional uncertainty and disempowerment responding to ethnic diversity in health care: a qualitative study. PLoS medicine. 2007;4(11):e323 Epub 2007/11/16. 10.1371/journal.pmed.0040323 . 18001148 PMC2071935

[30] Priebe S, Sandhu S, Dias S, Gaddini A, Greacen T, Ioannidis E, et al Good practice in health care for migrants: views and experiences of care professionals in 16 European countries. BMC public health. 2011;11:187 Epub 2011/03/29. 10.1186/1471-2458-11-187 . 21439059 PMC3071322

[31] Park ER, Betancourt JR, Kim MK, Maina AW, Blumenthal D, Weissman JS. Mixed messages: residents’ experiences learning cross-cultural care. Academic medicine: journal of the Association of American Medical Colleges. 2005;80(9):874–80. Epub 2005/08/27. . 16123471 10.1097/00001888-200509000-00019

[32] Pieper HO, MacFarlane A. "I’m worried about what I missed": GP registrars’ views on learning needs to deliver effective healthcare to ethnically and culturally diverse patient populations. Education for health (Abingdon, England). 2011;24(1):494 Epub 2011/06/29. . 21710416

[33] Weissman JS, Betancourt J, Campbell EG, Park ER, Kim M, Clarridge B, et al Resident physicians’ preparedness to provide cross-cultural care. Jama. 2005;294(9):1058–67. Epub 2005/09/08. 10.1001/jama.294.9.1058 . 16145026

[34] Rocque R, Leanza Y. A Systematic Review of Patients’ Experiences in Communicating with Primary Care Physicians: Intercultural Encounters and a Balance between Vulnerability and Integrity. PloS one. 2015;10(10):e0139577 Epub 2015/10/07. 10.1371/journal.pone.0139577 . 26440647 PMC4594916

[35] Carroll J, Epstein R, Fiscella K, Gipson T, Volpe E, Jean-Pierre P. Caring for Somali women: implications for clinician-patient communication. Patient education and counseling. 2007;66(3):337–45. Epub 2007/03/06. 10.1016/j.pec.2007.01.008 . 17337152 PMC3298771

[36] Rosenberg E, Leanza Y, Seller R. Doctor-patient communication in primary care with an interpreter: physician perceptions of professional and family interpreters. Patient education and counseling. 2007;67(3):286–92. Epub 2007/04/24. 10.1016/j.pec.2007.03.011 . 17448622

[37] Zendedel R, Schouten BC, van Weert JCM, van den Putte B. Informal interpreting in general practice: Are interpreters’ roles related to perceived control, trust, and satisfaction? Patient education and counseling. 2018;101(6):1058–65. Epub 2018/02/07. 10.1016/j.pec.2018.01.012 . 29402573

[38] Karliner LS, Jacobs EA, Chen AH, Mutha S. Do professional interpreters improve clinical care for patients with limited English proficiency? A systematic review of the literature. Health services research. 2007;42(2):727–54. Epub 2007/03/17. 10.1111/j.1475-6773.2006.00629.x . 17362215 PMC1955368

[39] Leanza Y, Boivin I, Rosenberg E. Interruptions and resistance: a comparison of medical consultations with family and trained interpreters. Social science & medicine (1982). 2010;70(12):1888–95. Epub 2010/04/10. 10.1016/j.socscimed.2010.02.036 . 20378224

[40] Brisset C, Leanza Y, Laforest K. Working with interpreters in health care: a systematic review and meta-ethnography of qualitative studies. Patient education and counseling. 2013;91(2):131–40. Epub 2012/12/19. 10.1016/j.pec.2012.11.008 . 23246426

[41] Hsieh E. Interpreters as co-diagnosticians: overlapping roles and services between providers and interpreters. Social science & medicine (1982). 2007;64(4):924–37. Epub 2006/11/28. 10.1016/j.socscimed.2006.10.015 . 17126465

[42] Charmaz K. Constructing grounded theory. Thousand Oaks, CA: Sage Publications; 2014.

[43] Glaser BG, Strauss AL. The discovery of grounded theory: strategies for qualitative research. New York: Aldine de Gruyter; 1967.

[44] Health care report 2017 [In Swedish]. Stockholm: The National Board of Health and Welfare, 2017 978-91-7555-411-2.

[45] The Health and Medical Services Act: SFS 1982:763 Stockholm: Ministry of Health and Social Affairs.

[46] Official Report of the Swedish Government. A more equal care is possible—an analysis of unwarranted differences in care, treatment and reception [In Swedish]. Stockholm: Swedish Agency for Health and Care Service Analysis, 2014 978-91-87213-29-8.

[47] Anell A, Glenngard AH, Merkur S. Sweden health system review. Health systems in transition. 2012;14(5):1–159. Epub 2012/08/17. . 22894859

[48] Burstrom B, Burstrom K, Nilsson G, Tomson G, Whitehead M, Winblad U. Equity aspects of the Primary Health Care Choice Reform in Sweden—a scoping review. Int J Equity Health. 2017;16(1):29 Epub 2017/01/29. 10.1186/s12939-017-0524-z . 28129771 PMC5273847

[49] Anell A. The public-private pendulum—patient choice and equity in Sweden. The New England journal of medicine. 2015;372(1):1–4. Epub 2015/01/01. 10.1056/NEJMp1411430 . 25551523

[50] The Primary Health Care Choice Reform [In Swedish]. Government proposition; 2008/09:29. Stockholm: The Swedish Government; 2008.

[51] The National Board of Health and Welfare. Statistics on Health Care Personnel. Official Statistics on the Number of Licensed Practitioners (2013) and their Labour Market Situation (2012) [in Swedish]. Stockholm: The National Board of Health and Welfare, 2015.

[52] Barbour RS, Kitzinger J. Developing focus group research: politics, theory and practice. London: SAGE; 1999.

[53] Bensing JM, Deveugele M, Moretti F, Fletcher I, van Vliet L, Van Bogaert M, et al How to make the medical consultation more successful from a patient’s perspective? Tips for doctors and patients from lay people in the United Kingdom, Italy, Belgium and the Netherlands. Patient education and counseling. 2011;84(3):287–93. Epub 2011/07/29. 10.1016/j.pec.2011.06.008 . 21795007

[54] Mazzi MA, Rimondini M, Boerma WG, Zimmermann C, Bensing JM. How patients would like to improve medical consultations: Insights from a multicentre European study. Patient education and counseling. 2016;99(1):51–60. Epub 2015/09/05. 10.1016/j.pec.2015.08.009 . 26337005

[55] Paternotte E, van Dulmen S, Bank L, Seeleman C, Scherpbier A, Scheele F. Intercultural communication through the eyes of patients: experiences and preferences. Int J Med Educ. 2017;8:170–5. Epub 2017/05/24. 10.5116/ijme.591b.19f9 . 28535143 PMC5457791

[56] Evans L, Trotter DR. Epistemology and uncertainty in primary care: an exploratory study. Family medicine. 2009;41(5):319–26. Epub 2009/05/07. . 19418279

[57] Ledford CJ, Cafferty LA, Seehusen DA. Socializing Identity Through Practice: A Mixed Methods Approach to Family Medicine Resident Perspectives on Uncertainty. Family medicine. 2015;47(7):549–53. Epub 2015/11/13. . 26562644

[58] Bhise V, Rajan SS, Sittig DF, Morgan RO, Chaudhary P, Singh H. Defining and Measuring Diagnostic Uncertainty in Medicine: A Systematic Review. Journal of general internal medicine. 2018;33(1):103–15. Epub 2017/09/25. 10.1007/s11606-017-4164-1 . 28936618 PMC5756158

[59] Fox RC. Medical uncertainty revisited. Handbook of social studies in health and medicine. 2000;409:425.

[60] Napier AD, Ancarno C, Butler B, Calabrese J, Chater A, Chatterjee H, et al Culture and health. Lancet. 2014;384(9954):1607–39. Epub 2014/12/03. 10.1016/S0140-6736(14)61603-2 . 25443490

[61] Inglehart R, C. Haerpfer, A. Moreno, C. Welzel, K. Kizilova, J. Diez-Medrano, M. Lagos, P. Norris, E. Ponarin & B. Puranen et al. (eds.). 2014. World Values Survey: Round 6—Country-Pooled. Datafile Version: http://www.worldvaluessurvey.org/WVSDocumentationWV6.jsp. Madrid: JD Systems Institute.

[62] Schwartz SJ, Unger JB, Zamboanga BL, Szapocznik J. Rethinking the concept of acculturation: implications for theory and research. The American psychologist. 2010;65(4):237–51. Epub 2010/05/12. 10.1037/a0019330 . 20455618 PMC3700543

[63] Dogra N. Cultural competence or cultural sensibility? A comparison of two ideal type models to teach cultural diversity to medical students. International Journal of Medicine. 2003;5(4):223–31.

[64] Neuliep JW. Intercultural communication: a contextual approach. Thousand Oaks, Calif;: SAGE; 2012.

[65] Foronda C, Baptiste DL, Reinholdt MM, Ousman K. Cultural Humility: A Concept Analysis. Journal of transcultural nursing: official journal of the Transcultural Nursing Society. 2016;27(3):210–7. Epub 2015/07/01. 10.1177/1043659615592677 . 26122618

[66] Tervalon M, Murray-Garcia J. Cultural humility versus cultural competence: a critical distinction in defining physician training outcomes in multicultural education. Journal of health care for the poor and underserved. 1998;9(2):117–25. Epub 1999/03/12. . 10073197 10.1353/hpu.2010.0233

[67] Muntinga ME, Krajenbrink VQ, Peerdeman SM, Croiset G, Verdonk P. Toward diversity-responsive medical education: taking an intersectionality-based approach to a curriculum evaluation. Adv Health Sci Educ Theory Pract. 2016;21(3):541–59. Epub 2015/11/26. 10.1007/s10459-015-9650-9 . 26603884 PMC4923090

[68] Powell Sears K. Improving cultural competence education: the utility of an intersectional framework. Medical education. 2012;46(6):545–51. Epub 2012/05/26. 10.1111/j.1365-2923.2011.04199.x . 22626046

[69] Stone L. Managing the consultation with patients with medically unexplained symptoms: a grounded theory study of supervisors and registrars in general practice. BMC Fam Pract. 2014;15:192 Epub 2014/12/06. 10.1186/s12875-014-0192-7 . 25477194 PMC4266896

[70] Glaser BG. Theoretical sensitivity: advances in the methodology of grounded theory. Mill Valley, Calif: Sociology Press; 1978.

[71] Bjork Bramberg E, Dahlberg K. Interpreters in cross-cultural interviews: a three-way coconstruction of data. Qualitative health research. 2013;23(2):241–7. Epub 2012/12/22. 10.1177/1049732312467705 . 23258420

[72] Ingvarsdotter K, Johnsdotter S, Ostman M. Lost in interpretation: the use of interpreters in research on mental ill health. The International journal of social psychiatry. 2012;58(1):34–40. Epub 2010/09/14. 10.1177/0020764010382693 . 20833705

